# Mesenchymal Stem Cell Exosomes as a New Strategy for the Treatment of Diabetes Complications

**DOI:** 10.3389/fendo.2021.646233

**Published:** 2021-04-29

**Authors:** Jiachao Xiong, Hao Hu, Rong Guo, Hui Wang, Hua Jiang

**Affiliations:** Department of Plastic Surgery, Shanghai East Hospital, Tongji University School of Medicine, Shanghai, China

**Keywords:** diabetes mellitus, complication, microvascular damage, mesenchymal stem cells, exosomes

## Abstract

Diabetes mellitus (DM) is a metabolic disease, now prevalent worldwide, which is characterized by a relative or absolute lack of insulin secretion leading to chronically increased blood glucose levels. Diabetic patients are often accompanied by multiple macrovascular complications, such as coronary heart disease, hypertension, macrovascular arteriosclerosis, and microvascular complications. Microvascular complications include diabetic kidney injury, diabetic encephalopathy, and diabetic foot, which reduce the quality of life and survival status of patients. Mesenchymal stem cell exosomes (MSC-Exos) possess repair functions similar to MSCs, low immunogenicity, and ease of storage and transport. MSC-Exos have been proven to possess excellent repair effects in repairing various organ damages. This study reviews the application of MSC-Exos in the treatment of DM and its common complications. MSC-Exos may be used as an effective treatment for DM and its complications.

## Introduction

Diabetes mellitus (DM) is a metabolic disease, now prevalent worldwide, characterized by chronic hyperglycemia, accompanied by disorders of glucose, adipose tissue, and protein metabolism, which are caused by defects in insulin secretion or action (1). The global prevalence of diabetes is continuously rising, perhaps due to social and economic development, changes in lifestyles, and population aging (2). DM is clinically common with type 1 and type 2 diabetes. Currently, type 1 diabetes is common in adolescents and requires lifelong insulin therapy because of the elimination of pancreatic islet β-cells by the immune response, resulting in an absolute lack of insulin secretion. Type 2 diabetes, commonly arising in elderly and obese patients, occurs due to the decreased insulin sensitivity of peripheral tissues, accompanied by defective insulin secretion in pancreatic islet β-cells (3, 4). Patients with type 2 diabetes mainly control blood glucose by taking drugs that increase the sensitivity of insulin target tissues or increase pancreatic islet β-cells, combined with individualized therapy, such as regulating blood lipids, controlling their body weight, and improving their lifestyle (5). Unfortunately, most patients with diabetes have unstable blood glucose levels. Long-term high levels of blood glucose can cause systemic macrovascular and microvascular damage, potentially leading to chronic complications in multiple tissues and organs, such as the eyes, kidneys, nerves, and heart, which is an important cause of cardiorenal failure, blindness, amputation, and even death (6). Therefore, the management of DM and its related chronic complications is particularly important.

Chronic diabetic patients often have multiple complications, such as diabetic kidney injury, diabetic encephalopathy, and diabetic foot, which reduce the quality of life and survival status of patients. Currently, there is no way to eliminate diabetes, and changes in patients’ lifestyles combined with drug therapy are the main ways to control blood glucose. However, the therapeutic methods for diabetes complications are insufficient and new methods are urgently required to improve the quality of life and survival rate of patients.

Mesenchymal stem cells (MSCs) are pluripotent progenitor cells that can differentiate into adipocytes, osteoblasts, chondrocytes, and other cell types of mesodermal origin (7). MSCs are characterized by their high self-renewal ability, low immunogenicity, and immune regulation ability, and play an important role in clinical cell therapy. MSCs originate from a wide range of sources and were first isolated from bone marrow (8). Subsequent studies have found that MSCs can be isolated from various human tissues, such as adipose tissue, umbilical cord, synovium, gingiva, menstrual blood, and urine (9, 10). The low immunogenicity of MSCs makes them a good material for transplantation. After transplantation, MSCs can chemoattract to the vicinity of damaged tissues and secrete a variety of growth factors and anti-inflammatory factors to promote the repair of damaged tissues (11). However, MSCs and mesenchymal tumor cells have numerous identical stem gene phenotypes, which strongly suggests that some early tumor cells are derived from MSCs (12). In addition, previous studies have found that MSCs promote tumor formation through vascularization, immune regulation, and the promotion of tumor interstitial remodeling (13, 14). These factors have greatly restricted the clinical applications of MSCs. Previous studies have found that exosomes isolated from MSC culture medium possess a repair function similar to MSCs and no risk of tumor formation (15, 16).

## Biological Functions of Mesenchymal Stem Cell Exosomes

**Figure 1:**
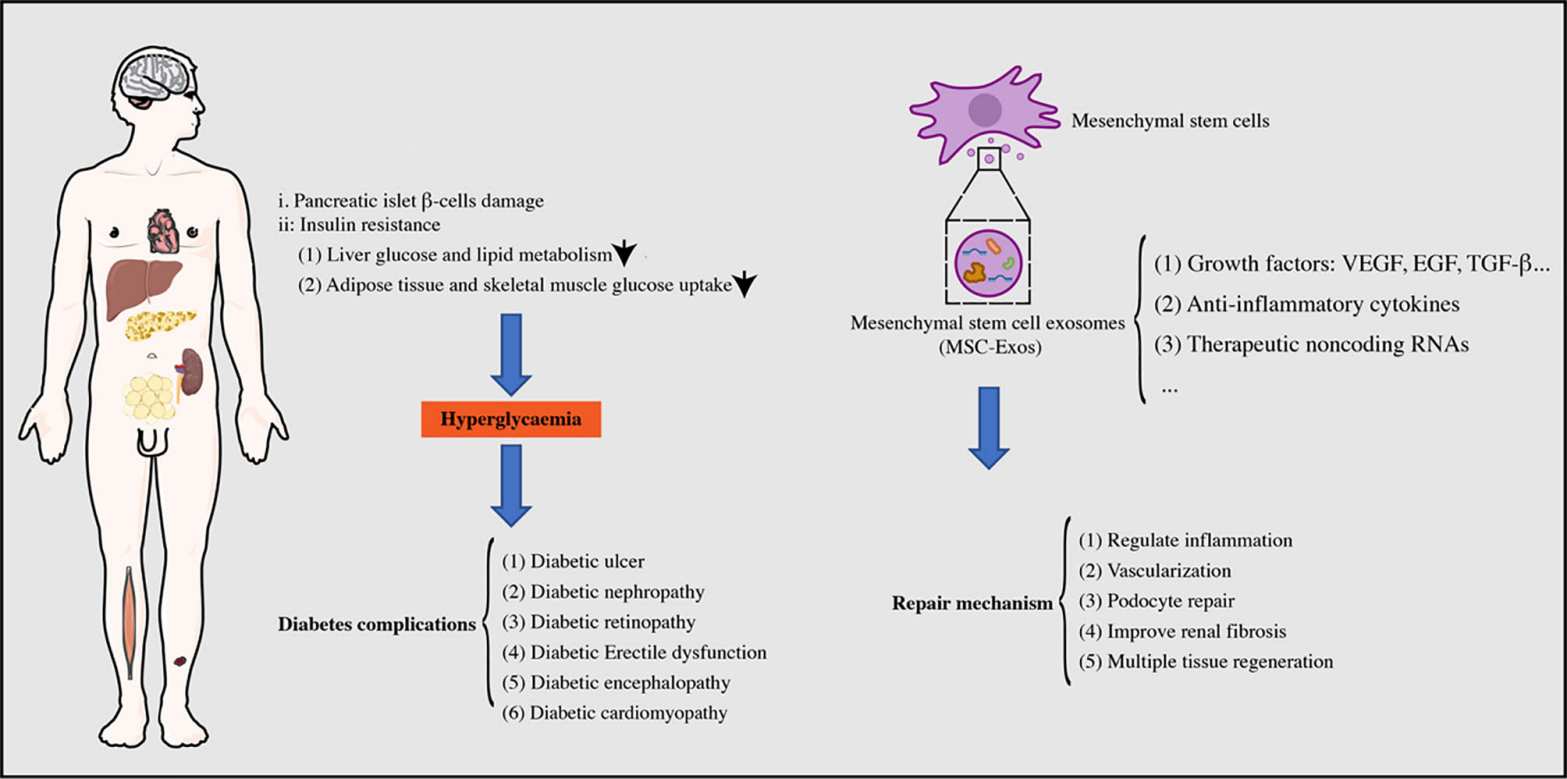
Diabetes/diabetes complications and mesenchymal stem cell exosomes repair.

MSC exosomes (MSC-Exos) are extracellular vesicles between 30–150 nm in diameter that have the same lipid bilayer structure as the cell membrane (17). Additionally, MSC-Exos possess more advantages than MSCs, such as lower immunogenicity, high stability, and easy storage (18). MSC-Exos contain multiple biologically active substances, such as lipids, proteins, and RNAs that can regulate the biological activities of target cells *via* membrane fusion or endocytosis (19, 20). Guo et al. (21) injected MSC-Exos into rats with spinal cord injury by intranasal administration and found that MSC-Exos greatly enhanced axon growth and angiogenesis, reduced the proliferation of microglia and astrocytes, and significantly promoted the repair of spinal cord injury. Moreover, MSC-Exos are rich in the C-C motif chemokine receptor-2 that promotes ischemia-reperfusion kidney injury healing by inhibiting macrophage function (22). MSC-Exos have shown excellent repair effects in various tissue injuries, such as liver, cardiovascular, and skin wounds that involve mechanisms of angiogenesis, regulation of cell proliferation, and immune regulation (17, 23, 24). The use of MSC-Exos, as an alternative to MSCs, has become a new strategy for tissue regeneration (**Figure 1**).

## Role of MSC-Exos in DM

Both type 1 diabetes and type 2 diabetes are accompanied by a certain degree of pancreatic islet damage. The progression of DM can be delayed by promoting islet regeneration and improving the sensitivity of insulin target tissues, which is a potential new treatment for diabetic patients (25) (**Table 1**).

**Table 1:** Mesenchymal stem cells (MSC) used in the treatment of diabetes mellitus (DM).

DM type	MSC types	Effect	Ref.
Type 1 diabetes	Menstrual blood MSC	Controlling blood glucose, increasing insulin level and promoting islet regeneration	(26)
	Adipose derived MSC		(27)
	Bone marrow MSC		(28)
Type 2 diabetes	Adipose derived MSC	Improve insulin resistance, improve liver sugar storage capacity, and promote islet regeneration	(29)
	Umbilical cord MSC		(30)
	Bone marrow MSC		(31)
	Umbilical cord MSC		(32)

Insulin deficiency in patients with type 1 diabetes occurs due to the autoimmune destruction of islet tissue, and MSC-Exos have the unique ability of immune regulation and can repair pancreatic islet tissue to alleviate DM. MSC-Exos can chemoattract pancreatic tissue and promote the regeneration of pancreatic β-cells and insulin secretion through the pancreatic and duodenal homeobox 1 pathway (26). Accumulating evidence has reported that MSC-Exos have a great therapeutic and regenerative effect on islet injury in type 1 diabetes by upregulating the levels of regulatory T cells, interleukin (IL)-4, IL-10, and transforming growth factor β (TGF-β), while downregulating IL-17 and interferon γ, ultimately improving the autoimmune response of diabetic mice and the regeneration of islets (27, 28).

Glucose transporter 4 transfer from intracellular vesicles to the plasma membrane is the final step of insulin signaling, in which the phosphorylation of insulin receptor substrate 1 and protein kinase B is an essential step, while the phosphorylation in type 2 diabetes patients is often blocked, leading to insulin resistance (33). Meanwhile, adenosine 5’-monophosphate (AMP)-activated protein kinase (AMPK) plays an important role in the regulation of glucose and lipid metabolism in skeletal muscle and liver, and is regarded as an important target to reverse type 2 diabetes-related metabolic abnormalities (34, 35). MSC-Exos can activate autophagy through the AMPK pathway or restore the phosphorylation of insulin receptor substrate 1 and protein kinase B, which contribute to muscle glucose transporter 4 expression to reverse peripheral insulin resistance; it also relieves the apoptosis of islet β-cells and restores the insulin secretion function of type 2 diabetes (30). Patients with type 2 diabetes are commonly associated with obesity (36, 37), which induces the M2 phenotype of macrophages through the transactivation of arginase-1 that promotes hepatic glucose and lipid metabolic balance to reduce obesity (29, 31, 32).

## Role of MSC-Exos in DM Complication

DM is the ninth most common cause of death globally, and most diabetic patients have at least one complication (38). A large observational study showed that 50% of patients with type 2 diabetes had microvascular complications, and 27% were associated with macrovascular complications (39). The development of common DM complications, including diabetic kidney injury, diabetic encephalopathy, and diabetic chronic ulcers, determines the quality of life of patients. In recent years, MSC-Exos have played a substantial role in the treatment of diabetic complications, providing a new approach for its treatment (**Table 2**).

**Table 2:** MSC used in the treatment of DM complication.

DM complication	MSC types	Effect/involved noncoding RNA	Ref.
Diabetes wound	Bone marrow MSC	Vascularization	(40)
	Adipose derived MSC	Vascularization	(41)
	Adipose derived MSC	Vascularization/miR-21-5p	(42)
	Bone marrow MSC	Vascularization	(43)
	Bone marrow MSC	Regulate inflammation/lncRNA H19	(44)
	Adipose derived MSC	Vascularization	(45)
	Synovium MSC	Vascularization/miR-126-3p	(46)
	Urine MSC	Vascularization	(47)
	Adipose derived MSC	Vascularization and regulate inflammation	(48)
	Induced pluripotent stem cell	Vascularization	(49)
	menstrual blood-derived MSC	Vascularization and regulate inflammation	(50)
	Adipose derived MSC	Vascularization	(51)
	Adipose derived MSC	Vascularization	(52)
	Adipose derived MSC	Vascularization/mmu_circ_0000250	(53)
	Bone marrow MSC	Vascularization and regulate inflammation	(54)
	Bone marrow MSC	Vascularization/miR-221-3p	(55)
	Umbilical cord MSC	Vascularization	(56)
Diabetic nephropathy	Adipose derived MSC	Podocyte repair/miRNA-215-5p	(57)
	Urine MSC	Podocyte repair	(58)
	Bone marrow MSC	Anti-fibrosis and promote renal function recovery	(59)
	Bone marrow MSC	Anti-fibrosis and promote renal function recovery	(60)
	Adipose derived MSC	Podocyte repair/miR-486	(61)
	Urine MSC	Podocyte repair/miR-16-5	(62)
	Umbilical cord MSC	Reduce kidney inflammation and improve kidney function	(63)
	Umbilical cord MSC	Reduce kidney inflammation and improve kidney function	(64)
Diabetic retinopathy	Adipose derived MSC	Retinal repair/miR-222	(65)
	Umbilical cord MSC	Retinal repair and regulation of inflammation/miR-126	(66)
Erectile dysfunction	Adipose derived MSC	Vascularization and anti-apoptosis	(67)
	Adipose derived MSC	Promote angiogenesis and anti-fibrosis/miR‐126, miR‐130a, miR‐132, miR‐let7b, miR‐let7c	(68)
	Adipose derived MSC	Vascularization and anti-inflammatory	(69)
	Bone marrow MSC	Vascularization and anti-inflammatory/miR-21-5p	(70)
Cognitive dysfunction	Bone marrow MSC	Nerve repair	(71)
	Bone marrow MSC	Nerve repair and anti-inflammatory/miR-146a	(72)
	Bone marrow MSC	Nerve repair	(73)
Diabetic stroke	Bone marrow MSC	Nerve repair/miR-9	(74)
	Bone marrow MSC	Nerve repair/miR-145	(75)
Submandibular gland dysfunction	Bone marrow MSC	Salivary gland function repair	(76)
Diabetic cardiomyopathy	MSC	Reduce myocardial injury and fibrosis	(77)

### Diabetes Ulcer

Wound healing consists of four overlapping and dynamic processes of hemostasis, inflammation, proliferation, and remodeling, and the obstruction of one of these steps leads to delayed healing (78). The healing of diabetic ulcers (DUs) is often blocked by factors, such as infection, peripheral neurovascular disease, hypoxia, and neuropeptide conduction, which increase the patient’s medical expenditure and prolong the hospitalization period. DUs, one of the most serious complications of DM, often occur on the feet of patients; therefore, they are also called diabetic foot. The incidence of DUs in patients with type 1 diabetes is approximately 20%, which is similar to that in patients with type 2 diabetes (79). In addition, DUs are an important cause of disability, and more than 15% of patients will undergo amputation (80). Despite the progress made in wound care, the United States Centers for Disease Control and Prevention reported that DUs are still the main cause of non-traumatic amputation (81). Therefore, it is necessary to improve the care and treatment of DUs. Similar to MSCs, MSC-Exos promote the healing of DUs by regulating the inflammatory microenvironment of the wound, promoting vascularization and anti-oxidant apoptosis (40).

#### Regulate Inflammation

Previous studies have shown that M1 macrophages can produce pro-inflammatory cytokines, such as IL-1β and tumor necrosis factor-alpha (TNF-α), while M2 macrophages can produce anti-inflammatory cytokines, such as TGF-β and VEGF (82, 83). Therefore, macrophage polarization plays a vital role in the regulation of the inflammatory response (84). Patients with diabetes are in a state of chronic inflammation, and the number of pro-inflammatory M1 macrophages in the damaged wound is significantly higher than that in anti-inflammatory M2 macrophages (85). Excessive polarization of M1 macrophages will inevitably increase the secretion of IL-1β, TNF-α, and other pro-inflammatory cytokines that prolong the inflammatory period of wound repair, which further leads to wound collagen regeneration and scar hyperplasia (86–88).

MSC-Exos increase the M2/M1 polarization ratio, which reduces the inflammation of DUs wounds and promotes healing (50). MSC-Exos inhibit the activation of the phosphatidylinositol 3-kinase/protein kinase B (PI3K/AKT) pathway and weaken the phosphorylation of AKT by promoting the expression of pentaerythritol tetranitrate (PETN) to regulate the M1/M2 polarization ratio. This inhibits the inflammatory response of wounds in diabetic rats and accelerates the rapid transition from the inflammation phase to the tissue regeneration phase (44, 54). It can be seen that the PETN/PI3K/AKT axis is closely related to macrophage polarization, which regulates the local inflammatory response and enhances the proliferation and migration ability of fibroblasts at the injury site that promotes the healing of DU wounds.

#### Vascularization

The degree of wound vascularization determines the healing rate and remodeling of the wound. Neovascularization occurs through the wound repair process. New capillaries are formed in granulation tissue after three days of injury; they grow rapidly and are interwoven into a network to provide oxygen and nutrients to the cells in the damaged area (89). In the process of angiogenesis, pro-angiogenic factors play a role in maintaining vascular growth. However, DM significantly reduces the level of pro-angiogenic factors, leading to blocked angiogenesis and delayed wound healing (90).

MSC-Exos are rich in multiple growth factors and therapeutic noncoding RNAs that can effectively promote the vascularization of skin wounds and are safer and more stable compared to cell therapy (91, 92). Studies have demonstrated that MSC-Exos are rich in circular RNAs, long noncoding RNAs, and microRNAs (miRNAs) that can regulate the expression of related repair genes to promote the vascularization and healing of DU wounds. Exosomes derived from mmu_circ_0000250-modified adipose-derived mesenchymal stem cells were found to promote the activation of autophagy to inhibit cell apoptosis in a high-glucose environment, promote the expression of SIRT1 through miR-128-3p adsorption, promote wound vascularization, and ultimately accelerate the healing of DM wounds (53). Yu et al. (55) found that MSC-Exos can promote the angiogenesis ability of endothelial cells; the expression of VEGF upregulates the expression of miR-221-3p and activates the AKT/endothelial nitric oxide synthase (eNOS) pathway to promote the vascularization of DM wounds. Moreover, MSC-Exos can be used as a good carrier of therapeutic noncoding RNA for the healing of DU wounds. Studies have applied miR-126-3p or miR-21-5p engineered MSC-Exos to diabetic wounds and found that they can activate the PI3K/AKT, mitogen-activated protein kinase (MAPK)/extracellular signal-regulated kinase (ERK), and Wnt/β-catenin pathways, which are closely related to blood vessel formation, to promote the vascularization and re-epithelialization of DM wounds and enhance the efficiency of healing (42, 46). In addition, compared with MSC-Exos alone, combining MSC-Exos with hydrogel materials to improve the survival rate of MSC-Exos applied to DU wounds has shown better vascularization and wound closure rates (41, 45, 51). Thus, MSC-Exos are expected to become a new biological agent for the treatment of DUs.

### Diabetic Nephropathy

Diabetic nephropathy (DN) is a severe type of microvascular kidney damage caused by DM that eventually develops into end-stage renal disease (ESRD), which is mostly characterized by a persistent protein or persistent glomerular filtration rate reduction. Once the course of DM exceeds 20 years, the incidence of DN can be as high as 35%, and approximately 8% of patients will progress to ESRD. Epidemiological statistics have shown that DM causes more than 40% of ESRD cases in the United States, and diabetes-related chronic kidney disease has also become the main cause of ESRD in hospitalized patients in China (38). Early DN manifests as glomerular hyperfiltration and microalbuminuria. As the disease progresses, a series of pathological changes appear in the kidneys, such as glomerular basement membrane thickening, mesangial expansion, glomerular sclerosis, podocyte loss, and renal interstitial fibrosis (93). The glomerular filtration rate gradually decreases and eventually develops into uremia (94). The various growth factors and therapeutic noncoding RNAs contained in MSC-Exos also have significant effects on improving renal function, delaying renal fibrosis, and repairing podocyte function, and are expected to become a new tool for the treatment of DN.

#### Podocyte Repair

Podocytes are an important part of the glomerular filtration barrier and are known to maintain barrier function together with vascular endothelial cells (95, 96). Studies have shown that hyperglycemia can induce podocyte apoptosis, which reduces the number of podocytes, resulting in proteinuria (97). Hence, preventing podocyte damage plays a vital role in the treatment of DN.

Studies have revealed that MSC-Exos have a significant protective effect against acute and chronic kidney injury (98, 99). MSC-Exos can effectively reduce podocyte damage induced by high glucose levels by delivering therapeutic miRNAs. MSC-Exos deliver therapeutic miRNAs, such as miR-215-5p, miR-486, miR-150, miR-134, and miR-16-5p to podocytes. This directly targets small mothers against decapentaplegic (Smad)-1 to weaken mammalian target of rapamycin (mTOR)-mediated autophagy or cooperates with vascular endothelial growth factor A (VEGFA) to protect podocytes from the effects of hyperglycemia, and improve the proliferation and migration of podocytes to protect renal function (57, 61, 62). In addition, exosomes from urine-derived stem cells were injected into a diabetic rat model through the tail vein, and it was observed that the overexpression of caspase-3 was inhibited, podocyte apoptosis was reduced, the proliferation of renal tubular endothelial cells was promoted, and the urine output and urinary microprotein excretion in DN rats were effectively reduced (58). These results indicate that MSC-Exos can alleviate podocyte injury and improve renal function recovery.

#### Improvement of Renal Fibrosis

Renal fibrosis is the central link in DN and ultimately leads to irreversible kidney damage. Renal fibrosis is closely related to inflammatory cell infiltration, epithelial-endothelial mesenchymal transition, and myofibroblast transdifferentiation. Inflammatory cells secrete multiple inflammatory cytokines, such as IL-1β, IL-6, TNF-α, and TGF-β1 (100, 101). In the early stage of DN, TGF-β1, an important inflammatory cytokine for renal fibrosis, interferes with the cell cycle and causes renal hypertrophy (102). Subsequently, TGF-β1 can activate the downstream Smad2/3, MAPKs, PI3K/AKT, RhoA, and Wnt/β-catenin signaling pathways to trigger the synthesis of the extracellular matrix and myofibroblast transdifferentiation, thereby accelerating the process of renal fibrosis (63).

Studies have reported that repeated administration of MSC-Exos to diabetic animal models can ameliorate glomerular hypertrophy, basement membrane thickening, and fibrosis, to reduce the progression of DN (59, 63, 64). MSC-Exos inhibit the secretion of TGF-β1 to reduce epithelial-endothelial mesenchymal transition and block the proliferation of mesangial cells induced by the MAPKs and PI3K/AKT/mTOR pathways, thus alleviating renal fibrosis (60). MSC-Exos contain growth cytokines, such as epidermal growth factor, fibroblast growth factor, hepatocyte growth factor, and VEGF, which have anti-inflammatory and anti-fibrotic effects. It can downregulate the expression of fibroblast markers, such as alpha-smooth muscle actin (α-SMA) and collagen IV in renal tubules, and improve renal fibrosis in DN rats.

### Diabetic Retinopathy

Diabetic retinopathy (DR) is an important cause of vision loss in the elderly. Hyperglycemia can cause multiple pathological changes in the retinal neurovascular unit, including optic nerve inflammation, glial hyperplasia, abnormal vascular permeability, and blood-retinal barrier decomposition, eventually leading to retinal fibrosis, vision loss, and blindness in severe cases (103–105). Epidemiology demonstrates that the prevalence of DR is as high as 28% in the United States and 25% in Asian countries (38). Thus, it is important to develop effective treatments for DR.

Previous studies have observed the activation of the NOD-like receptor family pyrin domain containing 3 (NLRP3) inflammasome, which leads to the maturation of proinflammatory cytokines, such as IL-1β, IL-18, and caspase-1 in the retinas of DM rats to mediate the apoptosis of retinal cells (106, 107). Previous studies (66) have found that MSC-Exos significantly downregulated the expression of high-mobility group box 1 (HMGB1), NLRP3, and NF-kappaB/P65 protein in DR rats, inhibiting the production of various inflammatory cytokines and reducing retinal vascular endothelial injury. The use of MSC-Exos rich in therapeutic noncoding RNAs may become a new method for the treatment and prevention of DR. MSC-Exos with overexpressed miR-126 was used to more effectively inhibit the activation of the HMGB1 signaling pathway and improve the inflammatory response in DR rats. In addition, Safwat et al. (65) found that MSC-Exos can deliver miR-222 to retinal cells and regulate signal transducer and activator of transcription 5A (STAT5) protein expression to inhibit neovascularization in advanced DR, which promotes retinal regeneration.

### Diabetic Erectile Dysfunction

Erectile dysfunction (ED) is a common chronic complication of DM and is defined as the inability to achieve or maintain an adequate erection during sexual intercourse (108). Epidemiology demonstrates that the prevalence of ED in men with diabetes is as high as 70% and is three times that of nondiabetic men (109). Previous studies reported that hyperglycemia caused VEGF signaling transduction, the synthesis of neuronal nitric oxide synthase and endothelial nitric oxide synthase to be blocked, and the level of oxygen free radicals to increase, which resulted in increased apoptosis of sponge endothelial cells and smooth muscle cells in ED patients (110, 111). However, the efficacy of oral phosphodiesterase type 5 inhibitors was unsatisfactory, and more effective methods need to be developed (112).

MSC-Exos injection therapy can significantly increase the ratio of intracavernosal pressure to mean arterial pressure and upregulate the expression of atrial natriuretic peptide, brain natriuretic peptide, and neuronal nitric oxide synthase to promote the recovery of erectile function in DM rats (69). MSC-Exos contain pro-angiogenic miRNAs (miR-126, miR-130a, and miR-132) and anti-fibrotic miRNAs (miR-let7b and miR-let7c), which may increase the proliferation of vascular endothelial cells and smooth muscle cells in the cavernous body by increasing the proliferation of vascular endothelial cells and the expression of smooth muscle markers (α-SMA) and anti-apoptotic proteins (Bcl-2) to alleviate ED (67, 68). In addition, MSC-Exos reduced apoptosis and promoted the proliferation of cavernous smooth muscle cells by delivering miR-21-5p to target programmed cell death 4, and significantly improved erectile function and smooth muscle density in DM rats (70).

### Diabetic Cardiomyopathy

Diabetic cardiomyopathy (DC) is the systolic and diastolic dysfunction caused by DM, which eventually leads to heart failure. Coronary artery disease and ischemic cardiomyopathy are the main contributors to cardiac death in diabetic patients (113). Early DC manifests as impaired diastolic function, but no significant changes in systolic function (normal ejection fraction). As the disease progresses, cardiac systolic function is affected by reduced ejection fraction, and the pathological manifestations include left ventricular hypertrophy and interstitial fibrosis (114). The heart is a terminally differentiated organ, and it is difficult to regenerate cardiomyocytes after damage (115). Exosomes derived from MSC therapy may be a new approach for DC repair.

MSC-Exos have shown good therapeutic effects against cardiac ischemic diseases. Exosomes derived from bone marrow MSCs were directly injected into rats with myocardial infarction, which increased the expression of the miR-19a/AKT/ERK axis by inhibiting PTEN; thus, myocardial cell apoptosis was reduced with the significant recovery of myocardial contractile function and the reduction of infarct size (116). Notably, MSC-Exos may have a protective effect against myocardial injury. Lai et al. (117) used human embryonic stem cell-derived MSC-Exos perfusion buffer in a mouse model of myocardial ischemia-reperfusion and observed that the activation of the AKT/ERK pathway and the inhibition of the c-Jun NH2-terminal kinase pro-apoptotic pathway in the myocardial tissue was accompanied by a significant improvement in cardiac function at 1 h, 48 h, and 28 days after intervention. MSC-Exos were injected into the tail vein of a rat model of diabetic myocardial injury and it was observed that MSC-Exos inhibited the TGF-β1/Smad2 signaling pathway to improve myocardial injury and fibrosis induced by DM (77). At present, there are few studies on MSC-Exos used in DC, but MSC-Exos has a powerful regulating and repairing effect on myocardial injury; therefore, the repair of DCs has great application prospects.

### Other Rare Complications

Other rare complications of DM include cognitive impairment, stroke, and submandibular gland dysfunction. Abnormal blood glucose metabolism results in central nervous system neuron damage, decreased hippocampal synaptic plasticity, astrocyte foot swelling, which leads to cognitive dysfunction, various vascular diseases and increased vascular permeability, which leads to ischemic stroke, and salivary gland function damage, which leads to salivary quality reduction and gland function disorder (71, 76). There are few reports on the application of MSC-Exos to the above-mentioned complications, but some curative effects have been achieved.

Studies have reported that MSC-Exos can act on damaged neurons and astrocytes to promote their repair and reverse cognitive dysfunction (72, 73). MSC-Exos can significantly reduce the expression of ATP-binding cassette A1 and type 1 insulin-like growth factor receptor by increasing the expression of miR-145 or reducing the expression of miR-9, and increasing the neurorepair and cognitive function improvement of stroke DM rats (74, 75). AbuBakr et al. (76) found that MSC-Exos inhibited the TGFβ signaling pathway through Smad2 and Smad3 to inhibit the damage of salivary glands caused by DM, which was manifested by the reduction of serum amylase and salivary IgA, which led to the restoration of salivary gland function.

## Conclusion

DM is a metabolic disorder prevalent worldwide, and its incidence is increasing annually. Long-term hyperglycemia causes many chronic complications with regards to microvascular disease, and there is a lack of effective treatment methods. MSC-Exos have a repair function similar to MSCs, but do not have the shortcomings of MSCs in terms of promoting tumor formation and the difficulties associated with storage. MSC-Exos are rich in a variety of growth cytokines, repair proteins, and therapeutic noncoding RNAs, which can promote the repair of organs damaged by DM and its complications by regulating inflammation, vascularization, and anti-apoptotic mechanisms. The use of MSC-Exos may be an effective treatment strategy for DM and its complications.

## Author Contributions

HJ and HW designed the study. JX and HH performed and drafted the manuscript. RG revised manuscript. All authors contributed to the article and approved the submitted version.

## Funding

This work was supported by the east hospital affiliated to Tongji University introduced talent research startup fund (grant number DFRC2019008) and the China Postdoctoral Science Foundation (grant number 2019M651587).

## Conflict of Interest

The authors declare that the research was conducted in the absence of any commercial or financial relationships that could be construed as a potential conflict of interest.
